# The effect of COVID-19 pandemic on depression and suicidal ideation in Korean community dwelling elderly

**DOI:** 10.1192/j.eurpsy.2023.327

**Published:** 2023-07-19

**Authors:** K. Kim, B.-H. Yoon, H. Jung, H. Yun

**Affiliations:** Naju National Hospital, Naju, Korea, Republic Of

## Abstract

**Introduction:**

The impacts of the coronavirus disease of 2019 (COVID-19) pandemic on mental health have been relatively severe.

**Objectives:**

This study examined the influence of the COVID-19 especially on depression and suicidal ideation in community-dwelling elderly in Korea.

**Methods:**

Data were employed from a survey on elderly mental health in Jeollanam-do (southwest province in Korea). A total of 2,423 elderlies were recruited from 22 counties in Jeollanam-do between April and October 2021. We used self-reported questionnaires, including sociodemographic factors, COVID-19 related stress, suicidal ideation, Geriatric Depression Scale-Short Form Korean Version (GDS-SF). Logistic regression was performed to examine the factors on depression and suicidal ideation

**Results:**

Of the 2423 subjects, 622 (25.7%) reported depressive symptoms and 518 (21.4%) reported suicidal ideation. The multivariate logistic regression analysis revealed that living alone, poor perceived health status, the worry of COVID-19 infection and restriction of daily activity due to COVID-19 pandemic were significantly associated with depression. Male sex, poor perceived health status, disability in house chores and depressive symptom are risk factors for suicidal ideation.

**Image:**

**Figure:**
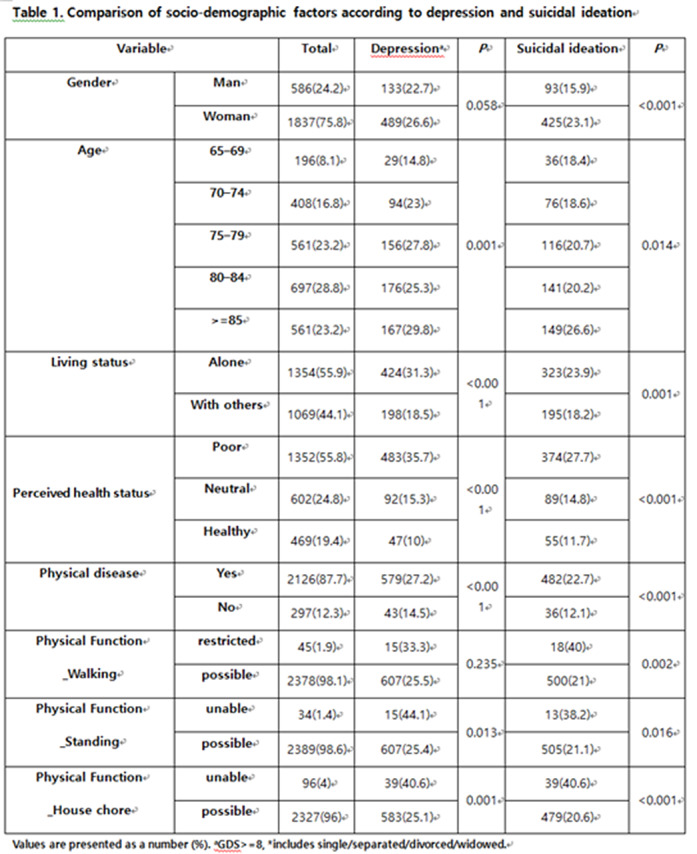

**Image 2:**

**Figure:**
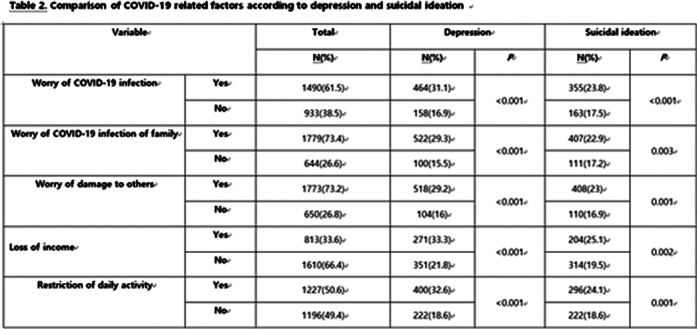

**Image 3:**

**Figure:**
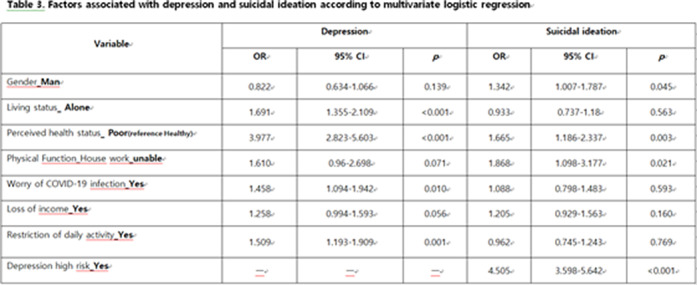

**Conclusions:**

These findings showed that increased risk factor for depression and suicidal ideation in community dwelling elderly during COVID-19 pandemic. We confirmed that feelings of isolation and negative perception of health were risk factors on depression in community dwelling elderly in the context of the COVID –19 pandemic. Also male, poor self-perceived health status, difficulty of independent living and worry and depression are increased the risk of suicidal ideation among the elderly.

**Disclosure of Interest:**

None Declared

